# Virulence genes expression profiling of different *Shigella flexneri* serotypes in response to sub-inhibitory concentrations of azithromycin and ciprofloxacin

**DOI:** 10.1186/s13099-022-00483-3

**Published:** 2022-02-22

**Authors:** Mehrzad Sadredinamin, Mahdi Shabani, Abdollah Karimi, Mohammad-Reza Sohrabi, Mohammadmahdi Karimi-Yazdi, Zohreh Ghalavand, Masoud Alebouyeh

**Affiliations:** 1 Department of Microbiology, School of Medicine, Shahid Beheshti University of Medical Sciences, Tehran, Iran; 2 Department of Immunology, School of Medicine, Shahid Beheshti University of Medical Sciences, Tehran, Iran; 3 Pediatric Infections Research Center, Research Institute for Children’s Health, Shahid Beheshti University of Medical Sciences, Tehran, Iran; 4 Social Determinants of Health Research Center, Shahid Beheshti University of Medical Sciences, Tehran, Iran; 5 Faculty of Paramedical Sciences, Mazandaran University of Medical Sciences, Sari, Iran

**Keywords:** *Shigella* spp., Serotypes, Sub-minimum inhibitory concentration, Gene expression profiling, Virulence factors, Drug resistance

## Abstract

**Background:**

Shigellosis is a self-limiting disease that antibiotic therapy could decrease its complications and duration. However, sublethal levels of antibiotics, may lead to alteration in disease state, besides its role in the emergence of resistant variants. To understand this link, we investigated diversity of *Shigella* serogroups in children with diarrhea, diversity of *S. flexneri* serotypes, cytotoxic potential, resistance patterns to antibiotics, and alteration in transcriptional expression of main virulence genes in response to sub-inhibitory concentrations of azithromycin and ciprofloxacin.

**Results:**

The most frequently isolated serogroups were *S. sonnei* (70.3%), followed by *S. flexneri* (29.1%) and *S. boydii* (0.6%). Ten serotypes were characterized among the *S. flexneri* isolates, including 2b, 1b, 2a, 1c, 4a, 3a, 3b, 6 and X and/or Xv. Antimicrobial susceptibility testing showed low frequency of multi-drug resistance phenotype among *S. flexneri* isolates with minimum inhibitory concentrations (MIC) of 0.5–64 and 0.25–8 µg/mL for azithromycin and ciprofloxacin, respectively. Gene expression analysis showed upregulation of *icsA* in serotype 4a after exposure with azithromycin, whereas other genes in the VirF pathway were downregulated, and downregulation of *virB* in serotypes 2a and 3a after exposure with ciprofloxacin, while upregulation of noted genes was detected.

**Conclusions:**

Alteration in transcription of key virulence genes of *S. flexneri* serotypes was shown in response to sublethal concentration of antibiotics. The detected incongruency in the extent of gene transcription proposed that diverse regulatory pathways are possibly mediating response to sub-MIC concentrations of antibiotics in *S. flexneri*.

## Introduction

*Shigella* is a common cause of diarrhea with high rates of mortality, especially in children younger than 5 years old [1]. The *Shigella* genus comprises four serogroups, including *S. boydii*, *S. dysenteriae*, *S. flexneri*, and *S. sonnei*, which *S. flexneri*, and *S. sonnei* are more common in clinical settings [2]. *S. flexneri* is considered as the predominant cause of shigellosis in developing countries. This serogroup is further classified into 19 serotypes, based on the O-antigen structure of lipopolysaccharide [3]. Diversity of these serotypes depend on the function of specific genes, which are responsible for the addition of glycosyl (*gtr*), acetyl (*oac*) or phosphoethanolamine (PEtN) to O-antigen different sugar residues and transportation of the repeat units from the cytoplasm to the periplasm (*wzx*) [4–6].

Although all *S. flexneri* serotypes use a similar pathogenic mechanism, some virulence factors are produced only by specific serotypes [7]. Essential virulence factors of *Shigella* spp. are located both on the chromosome and a large virulence plasmid. The plasmid-encoded genes play an important role in tissue invasion and the intracellular lifestyle of *Shigella* spp., which is activated via a regulatory cascade mediated by the plasmid-encoded VirF (Virulence factor production F) and VirB (Virulence factor production B) proteins. VirB in turn induces transcriptional expression of genes encoding the type III secretion system (*ipaB*-*D*) and effector proteins, (*ipaA*, *ipgB1*, *ipgD* and *icsB*) [8]. IcsA, which express and secret independently, enables actin-based motility for intracellular movement and cell-to-cell spreading in collaboration with *icsB* [9].

Although shigellosis is a self-limited disease, effective antibiotic therapy seems to shorten the symptom’s duration and prevent serious complications [10]. Resistance to antibiotics, such as ampicillin and trimethoprim-sulfamethoxazole, cause a change in treatment regimen in patients with shigellosis. Accordingly, azithromycin or ciprofloxacin were recommended by a number of international guidelines for the treatment of shigellosis in children [11]. Despite new reports about the emergence of resistance to these antibiotics in different countries, they are currently considered as drugs of choice in children [12–14]. Prescription of suboptimal dosage regimen could be responsible for emerging the resistant variants and change in the disease state [15]. Although there are increasing evidence showing that sub-minimal inhibitory concentrations (sub-MIC) of antibiotics can affect the expression of virulence factors in some bacteria, limited studies have evaluated the effect of sub-MIC concentration of antibiotics on *Shigella* [16]. This study was aimed to characterize *Shigella* serogroups in children with diarrhea, serotype diversity of *S. flexneri*, their cytotoxic potential, ability for interaction with cells, resistance patterns to antibiotics, and alteration in transcriptional expression of virulence genes *virB*, *ipaB*, *icsA* and *icsB* in response to sub-inhibitory concentration of azithromycin and ciprofloxacin.

## Results

### Patients and bacterial isolates

A total of 333 *Shigella* isolates, including *S. sonnei* (234, 70.3%), *S. flexneri* (97, 29.1%), *S. boydii* (2, 0.6%), and no *S. dysenteriae*, were recovered from children with diarrhea in Children’s Medical Center Hospital, Tehran, Iran. Among all the patients, 55.6% (185/333) were boys and (44.4%, 148/333) girls, ranging from 2 months to 14 years of age.

### Molecular serotyping of *S. flexneri* clinical strains

**Fig. 1:**
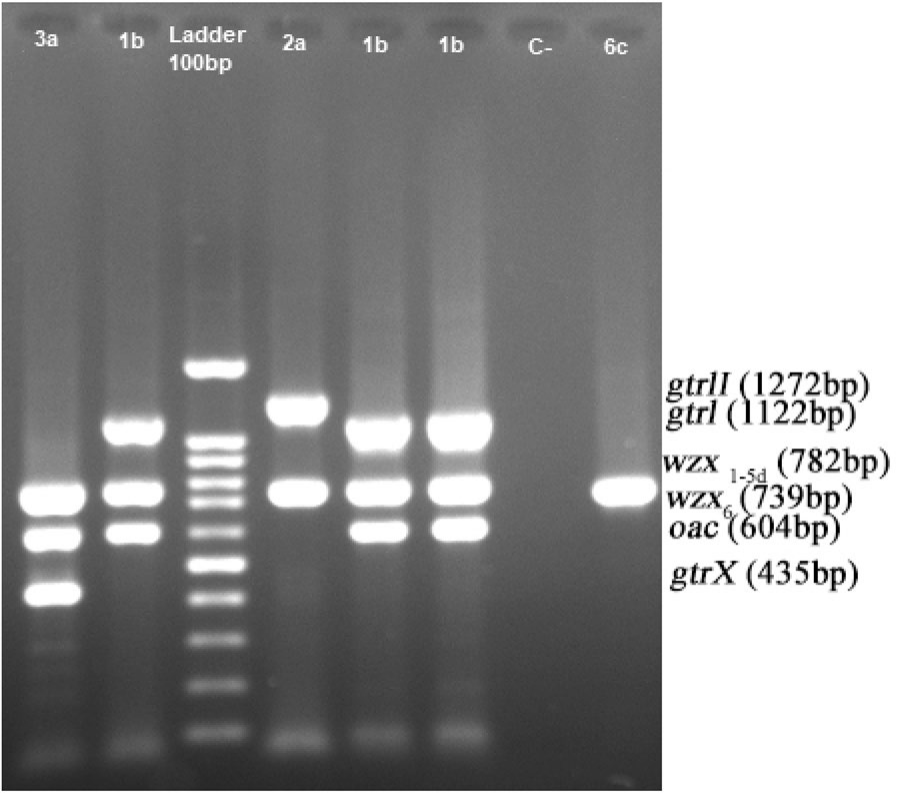
Multiplex PCR products of *S. flexneri* clinical strains. lane 1, *S. flexneri* serotype 3a; lane 4 *S. flexneri* serotype 2a, lanes 2, 5 and 6, *S. flexneri* serotype 1b; lane 3, Molecular size markers (100-bp DNA ladder; SinaClon); lane 4, *S. flexneri* serotype 2a; lane 7, Negative control.; lane 8, *S. flexneri* serotype 6c

Based on the Multiplex PCR results, 10 serotypes were found among 97 *S. flexneri* isolates (Fig. 1, Table 1). The most common serotypes were serotypes 2b and 1b (35.1% and 33%, respectively). As was shown in Table 2, a significant difference was detected for infection with different serotypes in different age groups. Accordingly, serotypes 1b and 1c infected children ≤ 5 and 6–10 years of age at higher frequency (71.9% and 50%, respectively), which was statistically significant (*p* = 0.037). No clinical isolates related to serotype 4b, 5a and 5b, which include gtrV, were characterized according to the Sun method [4]. GenBank accession numbers for the characterized genes are listed in Table 3.

**Table 1:** Distribution of serotypes among 97 *S. flexneri* isolates by multiplex PCR

Serotype	No	Gene loci								
		*Wzx1−5d*	*gtrI *	*gtrIc*	*gtrII*	*gtrIV*	*gtrV*	*gtrX*	*oac*	*wzx6*
1b	32	+	+	−	−	−	−	−	+	−
1c	8	+	+	+	−	−	−	−	−	−
2a	16	+	−	−	+	−	−	−	−	−
2b	34	+	−	−	+	−	−	+	−	−
3a	1	+	−	−	−	−	−	+	+	−
3b	1	+	−	−	−	−	−	−	+	−
4a	2	+	−	−	−	+	−	−	−	−
6	1	−	−	−	−	−	−	−	−	+
X or Xv	1	+	−	−	−	−	−	+	−	−
Y	1	+	−	−	−	−	−	−	−	−

Data are shown based on presence ( +) or absence (−) of the relevant genes as described by Sun et al. [4]

**Table 2:** Correlation between predominant serotypes of *S. flexnari* and age of patients

Serotype	Age of patients N^a^ (%)			*p *value
	≤ 5 years	6–10 years	11–14 years	
1b	23 (71.9)	7 (21.9)	2 (6.3)	0.037
1c	2 (25)	4 (50)	2 (25)	
2a	10 (62.5)	6 (37.5)	0 (0)	
2b	14 (41.2)	13 (38.2)	7 (20.6)	

^a^ N (%), Number and percentage of *S. flexneri* serotypes in different age groups

**Table 3:** PCR and real-time PCR primers used in this study

Target gene	Primer sequence (5' -3')	Product size (bp)	Accession number	References
*wzx1-5d*	F: CACTTGTTGGGTATGCTGG R: CCGGCAAACAGATTAGAAA	782	MN106906	[4]
*gtrI*	F: CTGTTAGGTGATGATGGCTTAG R: ATTGAACGCCTCCTTGCTATGC	1122	MN106907	[4]
*gtrIc*	F: AGGGAATGGCATTAGGGATCGG R: GCTGCAAGTGGTTTTTGTTGGA	518	MN106908	[4]
*gtrII*	F: ATTTATTGTTATTGGGGGTGGTTG R: ATTTGTTCTTTATTTGCTGGTT	1272	MN106909	[4]
*gtrIV*	F: ATGTTCCTCCTTCTTCCTTT R: TCCTGATGCTACCTTATCCA	378	MN099048	[4]
*gtrV*	F: AATACGATTCTCCTGGTGCTAAAC R: TAGGGCATTGCTTGTATCTTTCAT	905	–	[4]
*gtrX*	F: AATGCTGGATGGGATAATCACCTT R: GAGACGGCTTCTCCATGTTTTGCT	425	MN106910	[4]
*oac*	F: CTGTTCGGCTTTGAAAGTGCTG R: CGTAGGCGTACATAGCAAGCAAAGA	604	MN106911	[4]
*wzx6c*	F: TTAAGAGCGATCATTTC R: CCATCCAAGCGGACATT	739	MN919548	[4]
*16S rRNA*	F: AACGTCAATGAGCAAAGGTATTAA R: TACGGGAGGCAGCAGTGG	140	–	[39]
*virB*	F: GGAAGGGAGATTGATGGTAG R: GAACTTCAAGATCTGCTCCTGC	84	–	[39]
*ipaB*	F: CTGCATTTTCAAACACAGC R: GAGTAACACTGGCAAGTC	78	–	[39]
*icsA*	F: CTTTCGGGTACTCAAGAAC R: GAGAAAGTCCATCAACAGG	76	–	[39]
*icsB*	F: CTCAATTCAACACTCTTTCACAG R: GCTGTACCGATGCCATGAAAAC	82	–	[39] (This study)

bp, base pair; F, forward primer; R, reverse primer

### Antibiotic susceptibility profile

The results of antimicrobial susceptibility testing (AST) displayed that most of the isolates were resistant to ampicillin (99.0%), trimethoprim/sulfamethoxazole (89.7%), while resistance to cefotaxime (47.4%), cefepime (34%), nalidixic acid (30.9%), minocycline (28.9%), azithromycin (9.3%) and ciprofloxacin (7.2%) showed lower frequency (Table 4). multidrug resistant (MDR) pattern was detected in 15.5% of the isolates (15/97) (Table 5). Minimum inhibitory concentration (MIC) values for azithromycin and ciprofloxacin were in the range of 0.5–64 µg/mL and 0.25–8 μg/mL, respectively. The MIC_50_ and MIC_90_ were 2 and 0.5 μg/mL, and 4 and 8 μg/mL for azithromycin and ciprofloxacin, respectively (Table 6). Among all the MDR strains, one isolate belonging to serotype 4a was resistant to azithromycin; however, 4 isolates belonging to serotypes 2a (3/4 isolates) and 3a (1/4 isolate) were resistant to ciprofloxacin according to both disk diffusion and agar dilution results. A significant correlation was detected between infection with serotype 2a and resistance to ciprofloxacin (*p* = 0.001). There was no correlation between the serotypes and MDR phenotypes among the isolates (*p* = 0.764).

**Table 4:** Antimicrobial susceptibility of *S*. *flexneri* clinical isolates

Antimicrobial agents	*S. flexneri* clinical isolates			
	S	I	SDD	R
Penicillins				
Ampicillin	1 (1%)	0 (0%)	–	96 (99%)
Cephems				
Cefotaxime	49 (50.5%)	2 (2.1%)	–	46 (47.4%)
Cefepime	55 (56.7)	–	9 (9.3%)	33 (34%)
Folate pathway antagonists				
Trimethoprim-sulfamethoxazole	5 (5.2%)	5 (5.2%)	–	87 (89.7%)
Macrolides				
Azithromycin	87 (89.7%)	1 (1%)	–	9 (%9.3)
Quinolones and fluoroquinolones				
Ciprofloxacin	87 (89.7%)	3 (3.1%)	–	7 (7.2%)
Nalidixic acid	46 (47.4%)	21 (21.6%)	–	30 (30.9%)
Tetracyclines				
Minocycline	51 (52.6%)	18 (18.6%)	–	28 (28.9%)

Antimicrobial susceptibility test evaluated for antibiotics using Kirby–Bauer disk

Diffusion method (S, sensitive; SDD, susceptible, dose dependent; I, intermediate; R, resistant)

**Table 5:** Frequency and pattern of multidrug resistance phenotype among *S. flexneri* isolates in children with community acquired diarrhea

MDR phenotypes	Serotype 1b	Serotype 2a	Serotype 2b	Serotype 3a	Seroytpe 4a	Seroytpe X or Xv
	No. (%), n = 32	No. (%), n = 16	No. (%), n = 34	No. (%), n = 1	No. (%), n = 2	No. (%), n = 1
AMP/SXT/CIP/MN/NA	0	3 (30C)^a^	0	0	0	0
AMP/CTX/CPM/SXT/MN/NA	2	0	2	0	0	1
AMP/CTX/SXT/MN/NA/AZM	0	0	0	0	1 (19A)^a^	0
AMP/CTX/ SXT/CIP/NA	0	0	0	1 (3C)^a^	0	0
AMP/CTX/CPM/SXT/MN	1	0	3	0	0	0
AMP/CTX/SXT/MN	1	0	0	0	0	0

Multidrug-resistant (MDR) isolates are defined as those resistant to ≥ 3 classes among the third generation of cephalosporins, trimethoprim sulfamethoxazole, tetracycline, fluoroquinolones or macrolides

MDR, multidrug resistance; AMP, ampicillin; AZM, azithromycin; CPM, cefepime; CIP, ciprofloxacin; CTX, cefotaxime; MN, minocycline; NA, nalidixic acid; SXT, trimethoprim-sulfamethoxazole.

^a.^*S. flexneri* isolates 19A, 3C, and 30C with related MDR patterns were used for gene expression analysis

**Table 6:** The MIC results of MDR isolates of *S. flexneri* to azithromycin and ciprofloxacin

Antimicrobial agents	MIC^a^, µg/mL^a^																
	Azithromycin (0.25–128)								Ciprofloxacin (0.125–32)								
	0.5	1	2	4	8	16	32	64	0.125	0.25	0.5	1	2	4	8	16	32
N (%)	1 (6.66)	2 (13.33)	9 (60)	2 (13.33)	–	–	–	1 (6.66)	–	1 (6.66)	10 (66.6)	–	–	–	4 (26.6)	–	–
MIC_50_	2								0.5								
MIC_90_	4								8								

^a^ Minimum inhibitory concentration

N (%), number and percentage of *S. flexneri* isolates with different MIC values

Based on the above results, three MDR isolates of each serotype which were resistant to either azithromycin or ciprofloxacin were selected for studying the effect of sub-MIC concentration of azithromycin (serotype 4a) or ciprofloxacin (serotypes 2a and 3a) in infected cell culture.

### Selection of *S. flexneri* isolates for cell culture assay

Three serotypes, including 4a (19A), 3a (3C), and 2a (30C), were selected for cell culture assays based on resistance phenotype and MIC values to azithromycin and ciprofloxacin (64 µg/mL, 8 µg/mL and 8 µg/mL, respectively; Table 6).

### Cytotoxicity assay

**Fig. 2:**
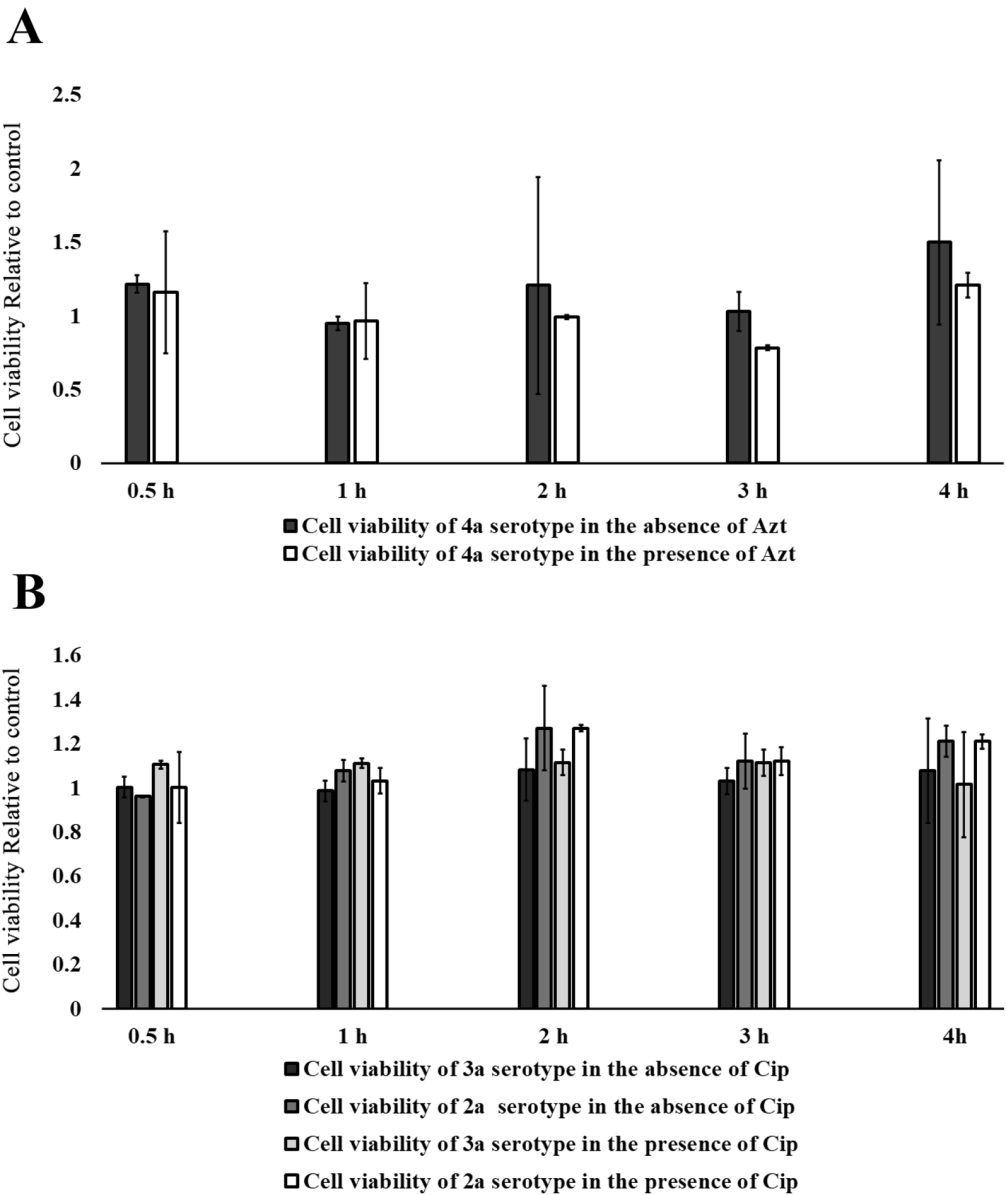
Viability of *S. flexneri* serotypes in the absence or presence of azithromycin and ciprofloxacin. Viability rates in HT-29 cells infected with different *S. flexneri* serotypes in the absence or presence of azithromycin (Azt, 32 µg/mL) (**A**) and ciprofloxacin (Cip, 4 µg/mL) (**B**) during 4 h. All the assays were done in duplicate. Data are expressed as the mean of replicates ± standard deviation of mean (std)

To determine appropriate time for analysis of changes in mRNA levels of *Shigella* virulence genes, HT-29 cells were infected with exponentially growing *S. flexenri* serotypes in the presence and absence of a sub-MIC concentration of azithromycin or ciprofloxacin and the viability of HT-29 cells were analyzed over 4 h. 3-(4,5-dimethyl-2-thiazolyl)-2,5-diphenyl-2H-tetrazolium bromide (MTT) results demonstrated that HT-29 infected cells produced low-grade cytotoxicity in the presence or absence of sub-MIC concentration of azithromycin or ciprofloxacin during the experiment (Fig. 2).

### Alteration in interaction with host cells

**Fig. 3:**
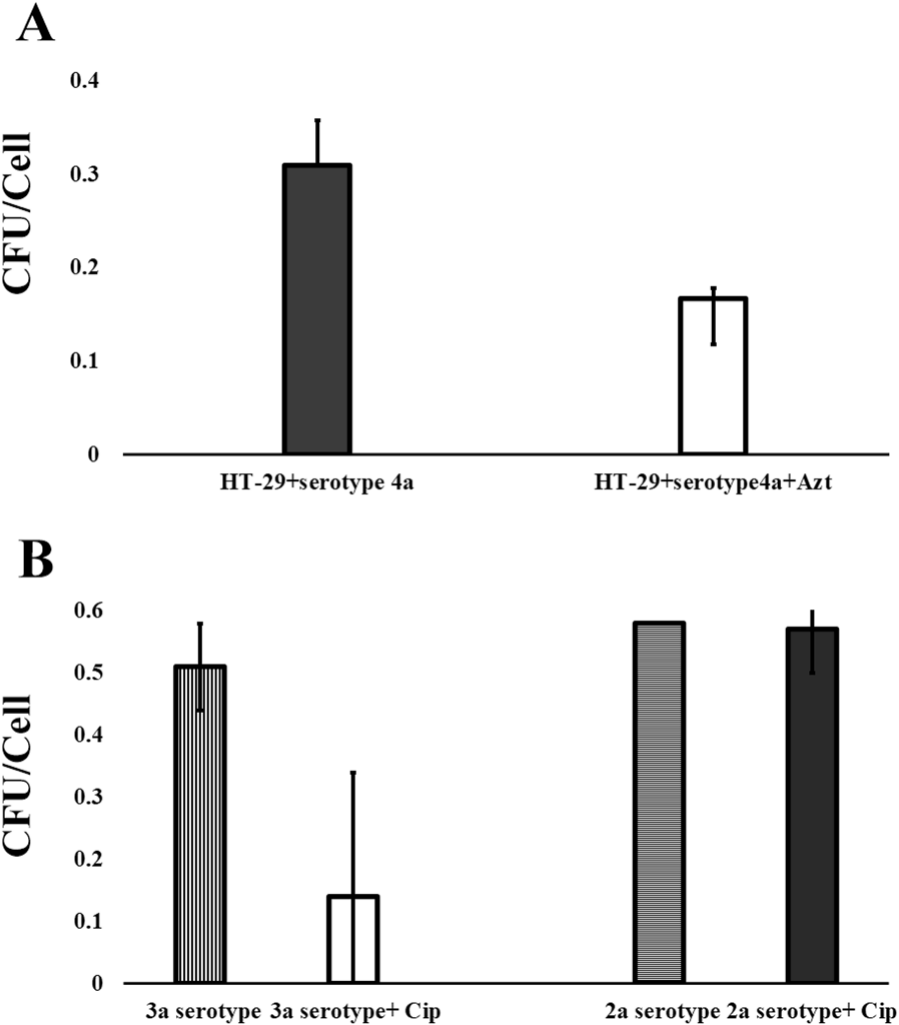
The ability for interaction of *S. flexneri* serotypes in the absence or presence of azithromycin and ciprofloxacin. The ability for interaction of *S. flexneri* serotypes was measured in the absence or presence of azithromycin (Azt, 32 µg/mL) (**A**) and ciprofloxacin (Cip, 4 µg/mL) (**B**) during 30 min. Data are expressed as mean of colony forming units (cfu)/cell ± std of HT-29 cell line (*10*^*5*^ Cells/well) from two independent experiments. Confluent cells in duplicate wells were inoculated with fresh cultures of *S. flexneri* strains at MOI of 100 (10^7^ cfu). The interaction rate for each assay was calculated based on number of grown colonies from cell lysates (CFU) on Muller Hinton Agar plates per each cell in tissue culture plates after treatment time

The effects of growth with sub-MIC concentration of antibiotics on the bacterial interaction are presented in Fig. 3. About 0.14 reduction in interaction of the treated strain with azithromycin (serotype 4a) compared with untreated ones was detected. In the case of serotypes 3a and 2a, which were treated with sub-MIC concentration of ciprofloxacin, a diversity was seen in the ability of interaction. Accordingly, 0.43 cfu/cell decrease in the interaction of serotype 3a after exposure to ciprofloxacin was detected compared with serotype 2a. The sub-MIC concentration of ciprofloxacin did not affect interaction of serotype 2a, remarkably (0.01 cfu/cell).

### Virulence gene expression profile of *S. flexneri* isolates in the presence and absence of ciprofloxacin or azithromycin

**Fig. 4:**
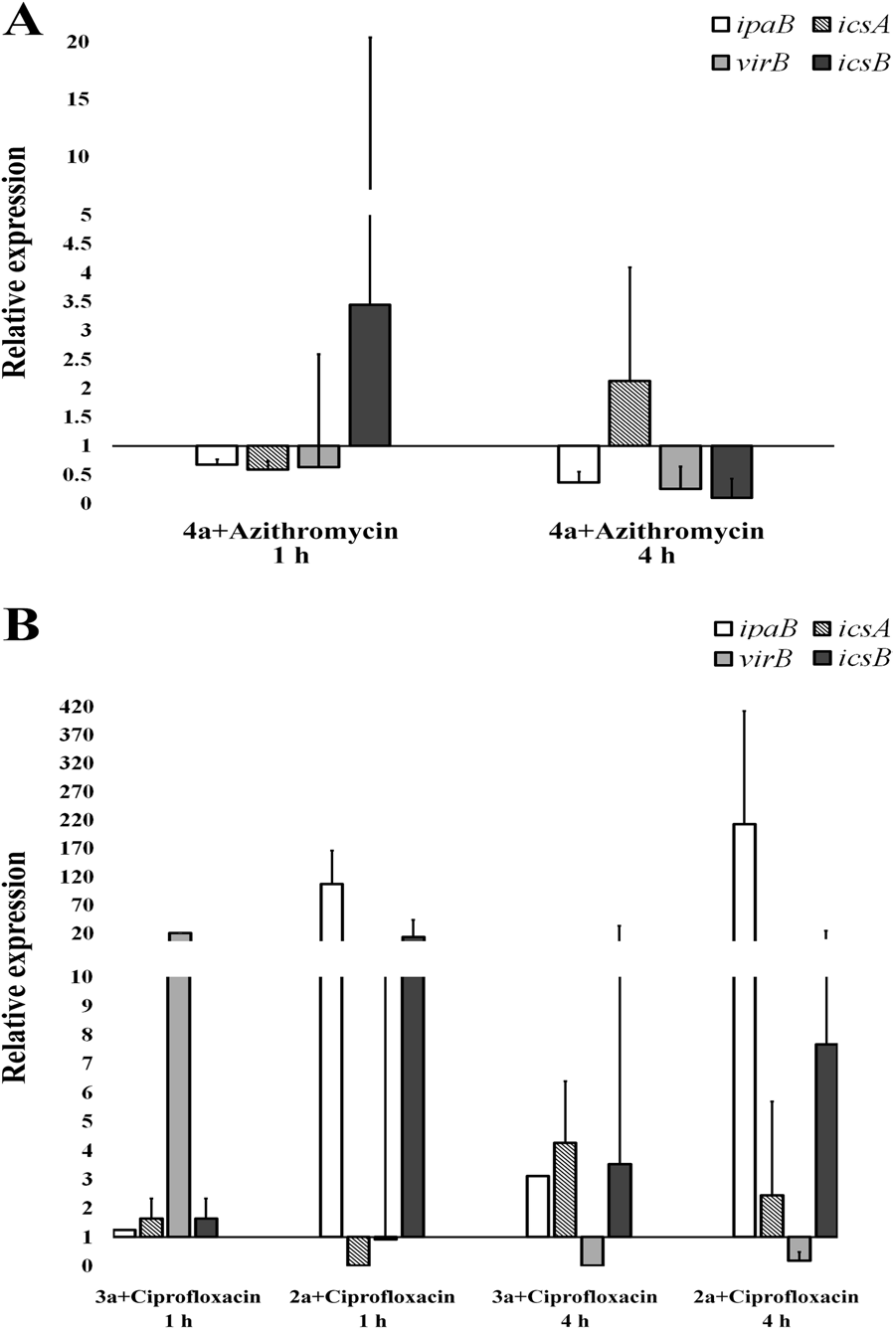
Diversity in transcriptional profiles of *S. flexneri* virulence genes in response to sublethal concentrations of antibiotics. Transcriptional profiles of invasion plasmid antigen (ipa) B, intracellular movement (ics) A, virulence factor production (vir) B and intracellular movement (ics) B genes of *S. flexneri* serotypes 4a, 3a and 2a after treatment with sub-MIC concentration of **A** azithromycin (32 µg/mL) or **B** ciprofloxacin (4 µg/mL) in HT-29 cell line. Expression levels of all genes were normalized to the levels of *16S rRNA* gene transcripts, and the folds of expression change were calculated using the relative comparison method (2^−ΔΔct^, RQ). Results shown are the mean ± std of two independent experiments

The relative virulence gene expression profiles were determined in HT-29 infected cells in the presence and absence of sub-MIC concentrations of azithromycin and ciprofloxacin (Fig. 4, Table 7).

**Table 7:** Alteration of gene expression in virulence genes of *S. flexneri* serotypes 4a, 3a and 2a

*S. flexneri* serotypes	Studied antibiotic classes	*virB*		*ipaB*		*icsA*		*icsB*	
		RQ values (t_1_–t_4_)							
		t_1_	t_4_	t_1_	t_4_	t_1_	t_4_	t_1_	t_4_
4a	Azithromycin	0.6285 ± 1	0.2526 ± 0.3	0.6736 ± 0.08	0.3686 ± 0.1	0.5864 ± 0.1	2.1214 ± 1.9	3.4462 ± 16	0.1005 ± 0.3
3a	Ciprofloxacin	21.112 ± 14.95	0.0103 ± 0.001	1.2527 ± 0.9	3.1167 ± 1.5	1.6472 ± 0.6	4.2575 ± 2.1	1.6472 ± 0.6	3.5186 ± 30.49
2a	Ciprofloxacin	0.9234 ± 14.9	0.178 ± 0.3	107.63 ± 58.88	213.04 ± 199	0.0229 ± 0.007	2.4538 ± 3.2	14.123 ± 30.24	7.6741 ± 17.19

Alteration in the transcription level of the virulence genes was analyzed in infected HT-29 cells with *S. flexneri* serotypes 4a, 3a and 2a at mRNA level after treatment with sub-MIC concentrations of azithromycin (32 µg/mL) or ciprofloxacin (4 µg/mL). Fold changes are expressed as RQ values (2^−ΔΔct^) by comparison of transcription levels between treated *vs* untreated cells at same conditions. Azithromycin (32 µg/mL) and ciprofloxacin (4 µg/mL) were used for treatment based on the estimated MIC values for each strain. All the assays were done in duplicate and results shown are the mean ± std of two independent experiments

VirB, virulence factor production (vir) B; IpaB, invasion plasmid antigen (ipa) B; IcsA intracellular movement (ics) A, IcsB intracellular movement (ics) B gene

We did not observed differences in expression level of the virulence genes except *virB* (21.1-fold increase) in serotype 3a followed by 1 hour exposure with ciprofloxacin (Fig. 4, Table 7).

Although transcriptional expressions of *ipaB* and *icsB* were upregulated after 1 hour exposure with ciprofloxacin in serotype 2a (107.6- and 14.1-fold increase, respectively), the *virB* transcription was remained unchanged while, the *icsA* was downregulated followed by 1 hour exposure with ciprofloxacin which was significant according to the paired *t* test method (0.2-fold decrease; *p* = 0.01).

The expression levels of *ipaB*, *icsA* and *icsB* genes were found to be upregulated in serotypes 2a and 3a after 4 h exposure with ciprofloxacin, but *virB* was downregulated (Fig. 4, Table 7). The observed changes in the expression levels of *ipaB* and *virB* were statistically significant for serotype 3a (*p* values of 0.03 and 0.001 for *ipaB* and *virB*, respectively).

Sub-MIC concentration of azithromycin, except for *icsB* (3.44-fold increase), showed no significant expression changes in serotype 4a, 1 h post-treatment. Azithromycin exposure for 4 h downregulated most of the virulence factors in serotype 4a, except for *icsA* (2.1-fold increase, Fig. 4, Table 7).

## Discussion

Shigellosis is one of the most common causes of morbidity among children less than 5 years of age [17]. *S. flexneri* is mainly responsible for the disease among children in the developing countries. However, serogroup transition was reported in some developing nations which is in line with our study [13, 18, 19].

*Shigella flexneri* consist of 19 serotypes [3], which may cause heterogeneity in the pathogenesis. To ascertain diversity of the isolated in symptomatic children in current study, we used molecular serotyping method [4]. The most frequent serotype was 2b (35.1%), followed by 1b, 2a, 1c, 4a, 3a, 3b, 6, X and Y. Molecular serotyping of *S. flexneri* was not done in previous studies in Iran, however, in a study by Khaghani et al. in 2014 using type-specific monovalent antisera, type 2 was detected in 50% (50/87) of *S. flexneri* isolates among children [20], which is comparable to our results (51.5%, including serotypes 2a and 2b). Our finding was also in consensus with the studies conducted in Pakistan and Bangladesh [21, 22], but in contradiction with previous studies where serotype 2a or 4c was reported as the most common serotype [23–25]. There is controversy among different studies to describe the second and third most common serotypes of *S. flexneri*. All these results suggest that heterogenous distribution of serotypes is likely, which could be associated with difference in time and geographical location.

In our study, serotype 1b was the most prevalent serotype among children less than 5 years (47%), while serotype 2b was common in children higher than 5 years of age (48.7%). In contrast to this finding, Vasilev et al. in 2003 reported serotype 2a and 6 as the most prevalent serotypes among children less than 5 years of age which was substituted with 1b in the older age groups [26].

Antibiotics, such as trimethoprim–sulfamethoxazole and ampicillin, were formerly prescribed for shigellosis; however, their restriction for administration was suggested by WHO due to widespread resistance [10]. In our study, all our *S. flexneri* strains were resistant to ampicillin and trimethoprim/sulfamethoxazole; however, low frequency of resistance to azithromycin and ciprofloxacin was detected that was comparable to previous reports [13, 27, 28]. This finding is consistent to recent recommendations for prescribing fluoroquinolones and azithromycin, as drug of choice for shigellosis in children [11, 29].

Excessive and indiscriminate use of antibiotics caused the emergence of MDR bacteria all over the world, which is a public health concern, since there are only a few effective antimicrobials available, especially for children [12, 15]. Our findings indicated MDR phenotype among a minority of *S. flexneri* isolates, which was lower than previous reports [13, 25, 30, 31]. This discrepancy might be attributed to the types of *Shigella* species, patients age range, isolation timing and geographical regions under study. The results also indicated that 13% of MDR isolates were serotypes 2b, 1b or 2a which was comparable with Nisa et al. study in Pakistan. However, this correlation was no significant in our study (*p* = 0.764). MIC distribution of azithromycin (MIC50 4 µg/mL) and ciprofloxacin (MIC50 0.5 µg/mL) in our MDR isolates was lower than previous reports [15, 16].

Previous results suggested that different classes of antibiotics can act as a chemical molecule and influence a range of processes, including virulence, at low concentrations [15]. This interaction could exert different outcomes in the strains presenting distinct resistance phenotypes. In a study was shown that sub-MIC concentration of norfloxacin could induce adaptive response of *S. sonnei* through downregulation of ribosomal proteins. The strain was sensitive to noted antibiotic with low MIC levels [16].

Although there is no other study on interaction of *Shigella* species with sub-MIC concentrations of antibiotics, study on *E. coli*, which shows 98.4% genomic identity with *Shigella* spp. supported similar interaction [32, 33]. A study by Bielaszewska et al. demonstrated that subinhibitory concentrations of ciprofloxacin induced shigatoxin production in enterohemorrhagic *Escherichia coli* (EHEC) serotypes [33].

VirF is considered as a master regulator of *Shigella* virulence pathway, which exert its effect through VirB and IcsA. According to the incongruency observed in transcriptional level of *icsA* (required for both adherence to cells and bacterial actin-based motility), which was upregulated in serotype 4a after 4 h exposure to azithromycin, while downregulation of *virB* and genes under its control (*ipaB* and *icsB*) was detected at same conditions in the present study, there seems to be another regulatory pathway. Previous studies on *Shigella* established that PhoP/PhoQ induction is able to positively regulate *icsA* transcription independent to *VirF* pathway [34]. PhoP/PhoQ seems to be a stress response regulatory pathway in bacteria. In a study, induction of PhoP/PhoQ two component system on *Salmonella enterica* serotype Typhimurium was demonstrated after exposure with sub-MIC concentration of nalidixic acid [35]. Hence overexpression of *icsA* presumably is the result of PhoP/PhoQ induction after exposure with azithromycin. Given that transcriptional expression of *virF* was not analyzed in this study, as well as incongruency in the extent of expression of *virB* compared with *ipaB* and *icsB* an hour after azithromycin exposure, the results again propose that *ipaB* and *icsB* transcription are not under *virB* control in response to sub-MIC concentration of azithromycin.

A similar pattern of transcriptional changes of *ipaB* and *icsB* was seen in serotypes 2a and 3a in response to sub-MIC concentration of ciprofloxacin. Accordingly, overexpression of these two genes occurred after 1 and 4 h post treatment, unlike serotype 4a that was downregulated while exposed to azithromycin at same conditions. This finding suggests that different regulatory pathways are involved in response to environmental stresses in *S. flexneri*. Further studies are needed to determine entity of these pathways and their mechanisms of regulation. Incongruency in transcription level of *virB* regulator compared with the *ipaB* and *icsB* (the genes under *virB* control) in response to ciprofloxacin, especially for serotype 2a, supported the existence of distinct *virF*/*virB* independent regulatory pathway for these virulence genes.

In addition, high level of gene expression in response to ciprofloxacin (Up to 213 folds) was a prominent finding, which may have impact on the pathogenicity of *Shigella* spp. and disease severity in patients.

In support of our findings, previous studies proved that sub-MIC concentrations of norfloxacin culminate the ppGpp (a stringent response molecule) levels, which upon its binding to DksA (as a regulatory protein), can directly results in upregulation of *ipaB*, *icsB*, *virF* and *icsA* in *S. flexneri*, while downregulate transcription of *virB* indirectly through Hfq [36, 37]. Although above data justify a discrepancy in *virB* expression compared with other investigated genes, the observed difference in transcriptional level of this gene at different time points (Serotype 3a, overexpression at 1 h post treatment with ciprofloxacin and down regulation after 4 h exposure) could suggest cooperation of distinct regulatory pathways for its regulation. To describe this difference a more comprehensive analysis of gene expression at transcriptomic and proteomic levels among different serotypes of *S. flexneri* is needed, which is considered as main limitation of our study. Lack of cytotoxic effect of the studied antibiotics at defined concentration and design of the study on HT-29 cell line (A colorectal cell line with similar feature to human intestine) suggested that similar interaction between *S. flexneri* and antibiotics can occur in patients upon administration of sublethal doses of antibiotics irrespective to level of MIC of responsible strains. Further studies are needed to establish importance of this interaction at clinical settings.

## Conclusions

In summary, our findings demonstrated a serogroup transition from *S. flexneri* to *S. sonnei* among children. While *S. flexneri* serotypes 2b and 1b were detected among children more frequently, it was shown that the infection can cause through 10 different serotypes in the studied population. A change in serotype from 1 to 2b was detected in children ≤  5 years when compared with those > 5 years of age. In line with other reports, the low incidence of resistance to therapeutic agents, including azithromycin and ciprofloxacin, among the *S. flexneri* isolates suggested that these antibiotics are still appropriate for treatment of shigellosis in children. However, continuous monitoring of antibiotic resistance patterns is required to control the prevalence of resistance in health care setting. Significant differences in mRNA expression patterns of *S. flexneri* virulence genes in response to sublethal concentration of azithromycin or ciprofloxacin was developed during 4 h exposure in a cell culture model. The observed incongruency for regulation of *ipaB*, *icsA*, *icsB*, and *virB* genes, suggests involvement of an alternative regulatory pathway independent to *virF* in response to sub-MIC concentrations of antibiotics. These results necessitate extending our knowledge of the effect of sub-MIC concentrations of antibiotics on other virulence associated genes and involved regulatory pathways for better management of shigellosis in children.

## Materials and methods

### Study design and bacterial isolates

The present study was conducted on clinical *Shigella* strains collected from children with acute diarrhea who referred to the Children’s Medical Center, Tehran, Iran from March 2016 to September 2018. This study was approved by the local ethics committee of Shahid Beheshti University of Medical Sciences (IR.SBMU.MSP.REC.1397.566).

Isolates were biochemically identified and serogroups were determined by using slide agglutination tests with serogroup specific antisera (Statens serum institute, Denmark). Confirmed isolates were stored in Tryptic Soy broth (TSB) (Merck, Germany) with 15% glycerol at − 70 °C for further analysis.

### Molecular serotyping

#### DNA preparation

To obtain genomic DNA, *S. flexneri* strains were grown overnight on Mueller–Hinton agar (Merck, Darmstadt, Germany), then in order to recover fresh culture a single colony of each sample was selected from MHA and inoculated in LB media at 37 °C overnight. DNA was extracted for further molecular analysis using a High Pure PCR Template Preparation kit (Roche Co., Germany) according to the manufacturer's instructions.

#### Serotyping by multiplex PCR assay

Multiplex PCR assay was carried out for molecular serotyping of *S. flexneri* using specific primers synthesized by Bioneer Company (Cheongju, South Korea) (Table 3, Fig. 1). Except *wzx6c*, indicative of serotype 6, which was characterized by singleplex PCR, all genes were detected in a total volume of 30 µL, containing DNA template (50 ng), each primer (10 µM), distilled water and 2× master mix with 2 mM MgCl_2_ final concentration. (Ampliqon, Denmark).

The thermal profile for amplification comprised an initial denaturation for 15 min at 95 °C, followed by 30 cycles of denaturation at 94 °C for 30 s, annealing at 55 °C for 30 s and elongation at 72 °C for 1 min and a final elongation for 10 min at 72 °C.

### Antimicrobial susceptibility testing

#### Disk diffusion

AST was conducted by using Kirby–Bauer disk diffusion method on Muller-Hinton Agar (MHA) (Merck Co., Darmstadt, Germany) according to Clinical Laboratory and Standards Institute (CLSI; 2018) guidelines and *Escherichia coli* ATCC 25922 was used as a control strain. The commercial antibiotics (Mast, England) used in the study included ampicillin (AP, 10 μg), cefotaxime (CTX, 30 μg), cefepime (CPM, 30 μg), trimethoprim/sulfamethoxazole (SXT, 1.25/23.75 μg), ciprofloxacin (CIP, 5 μg), minocycline (MIN, 30 μg), nalidixic acid (NA, 30 μg) and azithromycin (AZM, 15 μg).

Fisher's Exact Test was used to analyze correlation of *S. flexneri* serotypes with antibiotic resistance patterns and age groups. Two-sided *p* value of < 0.05 was considered statistically significant.

#### Minimum inhibitory concentration (MIC) assay

The MICs of azithromycin and ciprofloxacin were determined by agar dilution method according to Clinical Laboratory and Standards Institute (CLSI; 2018) guidelines for the MDR *S. flexneri* isolates.

Stock solutions were prepared from azithromycin in 95% ethanol (Rouz Darou, Iran) and ciprofloxacin in distilled water (Rouz Darou, Iran) to provide concentration ranging from 0.25 µg/mL to 128 µg/mL for azithromycin and concentration ranging from 0.125 µg/mL to 32 µg/mL for ciprofloxacin. *S. flexneri* ATCC 12,022 was used as a quality control.

### Cell culture

#### HT-29 infection protocol

A similar infection protocol was used for all cell culture assays. Briefly, HT-29 cells (Pasteur institute of Iran) were cultured in tissue culture flasks containing RPMI 1640 medium supplemented with 10% fetal bovine serum, 1% non-essential amino acids. The monolayer was used for MTT assay, interaction assay, and gene expression analysis one week after confluency. The monolayer was infected with three defined *S. flexneri* serotypes, 2a, 3a, and 4a, at a multiplicity of infection of 100 (MOI 100) in the presence or absence of Sub-MIC concentrations of ciprofloxacin (4 µg/mL) or azithromycin (32 µg/mL), in all the experiments. All the assays were done in duplicate.

#### Cytotoxicity assay

The effect of *S. flexneri* serotypes on HT-29 cytotoxicity in the presence and absence of Sub-MIC concentration of ciprofloxacin or azithromycin was determined via MTT assay. Prior to MTT assay, HT-29 cells were seeded at 10^4^ cell/well into 96-well plates and incubated overnight at 37 °C. The monolayer was then infected with the *S. flexneri* serotypes as mentioned above and incubated at 37 °C for 30 min, 1, 2, 3 and 4 h. MTT assay was done according to the instruction protocol using Kalazist kit (Tehran, Iran). Absorbance was measured at 570 and 690 nm by ELISA reader 4 h after addition of the MTT solution. Viability was measured according to the following formula:

$${\text{Cell viability ratio}} = {\text{sample absorbance/control absorbance}}$$

#### Altered interaction of *S. flexneri* with cells after antibiotics treatment

To compare changes in the interaction of different *Shigella* serotypes in the presence and absence of the antibiotics, the HT-29 cells were seeded into 24-well plates at a density of ∼10^5^ cells per well. The cell monolayer was then infected with *S. flexneri* serotypes as described above. After 30 min incubation, the infected cells were washed three times with Phosphate-buffered saline (PBS) and lysed with 1% Triton X-100 in PBS. Number of colonies per microliter of the lysate was enumerated by 10 times serial dilution on Mueller Hinton agar medium after 20 h incubation at 37 ˚C. The ability for interaction was measured as mean of cfu/cell in two independent experiments. The paired *t* test was used for statistical analysis.

### Gene expression analysis

#### RNA extraction and reverse transcription-PCR analysis

The HT-29 cells were seeded into 6-well plates at density of 5 × 10^4^ cells/well and cultured at 37 °C for 24 h. The monolayer was infected with *S. flexneri* serotypes as described above. Following 1 and 4 h incubation, the monolayer was washed three times with PBS, pH 8. Trypsinization of the monolayer was done at the two times intervals. The prepared lysates were stored at − 70 °C until RNA extraction. Total RNA from the infected cells were extracted with BioFACT ^™^ Total RNA Prep Kit (Biofact, South Korea), as described by the manufacturer instruction. DNase treatment was done using the RNase-free DNase Set (Sinaclon, Tehran, Iran) and RNA extracts were frozen at −70 °C until use. After adjusting RNA concentrations (85 ng) and assessment of reverse transcription efficacy using different primers, cDNA synthesis was done at 47 °C (*ipaB* and *icsB*) and 50 °C (*virB*, *icsB* and *16srRNA*) using the Superscript II First-Strand Synthesis System with specific primers in two separate reactions Table 3, as described by the manufacturer (Biofact, South Korea). *16S rRNA* was served as endogenous internal control gene in this experiment.

#### Quantitative real-time PCR

To determine the relative fold changes of *virB*, *ipaB*, *icsA* and *icsB* among HT-29 cells treated with different strains of *S. flexneri* serotypes in the presence and absence of Sub-MIC concentrations of ciprofloxacin (4 µg/mL) or azithromycin (32 µg/mL), relative quantitative (RQ) real-time PCR was performed by a Rotor-Gene 6000 (Corbett Research, Sydney, Australia). For each reaction, 13.5 μL of SYBR Green PCR Master Mix (Ampliqon, Denmark), 2 μL of cDNA (170 ng) and 1 μL of each primer and 8.5 µL of distilled water were used. The thermal cycling conditions were composed of initial denaturation at 95 ºC for 15 min, followed by 40 cycles at 95 ºC for 30 s, 57 ºC for 30 s and 72 ºC for 30 s. All the experiments were conducted in duplicates at two independent conditions. Moreover, the internal replicates within each assay were also used for all experiments.

In this study, all the expression analysis was done in duplicate. Accordingly, each treatment was done in two separate wells and RNA extracts from both wells were used for Real-time PCR. RNA extracts of untreated wells were used as reference for calculation of fold changes in transcription of target genes. The relative quantification (RQ) in gene expression was determined by analysis of the changes in geometric mean of the Ct values for each strain after treatment compared to mean Ct values of untreated ones. Accordingly, 2^−ΔΔct^ formula was used and fold changes ≥ 2 and  ≤ 0.5 were considered significant as described by Hu et al. [38]. Significance level was analyzed by paired *t* test.

## Data Availability

Not applicable.

## References

[1] Kotloff KL, Riddle MS, Platts-Mills JA, Pavlinac P, Zaidi AK (2018). Shigellosis. Lancet.

[2] Talukder KA, Islam MA, Khajanchi BK, Dutta DK, Islam Z, Safa A (2003). Temporal shifts in the dominance of serotypes of *Shigella* dysenteriae from 1999 to 2002 in Dhaka Bangladesh. J Clin Microbiol.

[3] Brengi SP, Sun Q, Bolaños H, Duarte F, Jenkins C, Pichel M (2019). PCR-based method for *Shigella flexneri* serotyping: international multicenter validation. J Clin Microbiol.

[4] Sun Q, Lan R, Wang Y, Zhao A, Zhang S, Wang J (2011). Development of a multiplex PCR assay targeting O-antigen modification genes for molecular serotyping of *Shigella flexneri*. J Clin Microbiol.

[5] Brengi SP, Sun Q, Bolaños H, Duarte F, Jenkins C, Pichel M (2019). PCR-based method for *Shigella flexneri* serotyping: international multicenter validation. J Clin Microbiol.

[6] Gentle A, Ashton PM, Dallman TJ, Jenkins C (2016). Evaluation of Molecular methods for serotyping *Shigella flexneri*. J Clin Microbiol.

[7] Barry EM, Pasetti MF, Sztein MB, Fasano A, Kotloff KL, Levine MM (2013). Progress and pitfalls in *Shigella* vaccine research. Nat Rev Gastroenterol Hepatol.

[8] Schroeder GN, Hilbi H (2008). Molecular pathogenesis of *Shigella* spp.: controlling host cell signaling, invasion, and death by type III secretion. Clin Microbiol Rev.

[9] Mattock E, Blocker AJ (2017). How do the virulence factors of *Shigella* work together to cause disease?. Front Cell Infect Microbiol.

[10] World Organization Health. Guidelines for the control of shigellosis, including epidemics due to *Shigella* dysenteriae type 1. 2005.

[11] Williams PC, Berkley JA (2018). Guidelines for the treatment of dysentery (shigellosis): a systematic review of the evidence. Paediatr Int Child Health.

[12] Darton TC, Tuyen HT, Newton PN, Dance DA, Phetsouvanh R, Davong V (2018). Azithromycin resistance in *Shigella* spp.Southeast Asia. Antimicrob Agents Chemother.

[13] Karimi-Yazdi M, Ghalavand Z, Shabani M, Houri H, Sadredinamin M, Taheri M (2020). High rates of antimicrobial resistance and virulence gene distribution among *Shigella* spp. isolated from pediatric patients in Tehran Iran. Infect Drug Resist.

[14] Gruninger RJ, Johnson RA, Das SK, Nelson EJ, Spivak ES, Contreras JR (2017). Socioeconomic determinants of ciprofloxacin-resistant *Shigella* infections in Bangladeshi children. Pathog Immun.

[15] Andersson DI, Hughes D (2014). Microbiological effects of sublethal levels of antibiotics. Nat Rev Microbiol.

[16] Chi WB, Zoqratt MZHM, Gan I, Ayub Q, Siew TH (2020). A fluoroquinolone-sensitive *Shigella* sonnei UKMCC1015 downregulates the expression of ribosomal proteins in response to sub-lethal concentration of norfloxacin. BioRxiv.

[17] Alemu A, Geta M, Taye S, Eshetie S, Engda T (2019). Prevalence, associated risk factors and antimicrobial susceptibility patterns of *Shigella* infections among diarrheic pediatric population attending at Gondar town healthcare institutions, Northwest Ethiopia. Trop Dis Travel Med Vaccines.

[18] Vinh H, Nhu NTK, Nga TVT, Duy PT, Campbell JI, Hoang NVM (2009). A changing picture of shigellosis in southern Vietnam: shifting species dominance, antimicrobial susceptibility and clinical presentation. BMC Infect Dis.

[19] Bangtrakulnonth A, Vieira AR, Lo Fo Wong DM, Pornreongwong S, Pulsrikarn C, Sawanpanyalert P (2008). *Shigella* from humans in Thailand during 1993 to 2006: spatial-time trends in species and serotype distribution. Foodborne Pathog Dis.

[20] Khaghani S, Shamsizadeh A, Nikfar R, Hesami A (2014). *Shigella flexneri*: a three-year antimicrobial resistance monitoring of isolates in a Children Hospital, Ahvaz Iran. Iran J Microbiol.

[21] Nisa I, Haroon M, Qasim M, Driessen A, Nijland J, Yasin N (2020). Association of serotype with antimicrobial resistance patterns among *Shigella flexneri* isolates from Pakistan: the importance of serotype 2b. Pediatr Infect Dis J.

[22] Talukder KA, Dutta DK, Safa A, Ansaruzzaman M, Hassan F, Alam K (2001). Altering trends in the dominance of *Shigella flexneri* serotypes and emergence of serologically Atypical *S. flexneri* strains in Dhaka Bangladesh. J Clin Microbiol.

[23] Das A, Mandal J (2019). Extensive inter-strain diversity among clinical isolates of *Shigella flexneri* with reference to its serotype, virulence traits and plasmid incompatibility types, a study from south India over a 6-year period. Gut Pathogens.

[24] Qu M, Zhang X, Liu G, Huang Y, Jia L, Liang W (2014). An eight-year study of *Shigella* species in Beijing, China: serodiversity, virulence genes, and antimicrobial resistance. J Infect Dev Ctries.

[25] Wang Y, Ma Q, Hao R, Zhang Q, Yao S, Han J (2019). Antimicrobial resistance and genetic characterization of *Shigella* spp. in Shanxi Province, China, during 2006–2016. BMC Microbiol.

[26] Vasilev V, Andorn N, Japheth R, Agmon V (2004). Variability of *Shigella flexneri* serotypes in Israel during a period of two years: 2000 and 2001. Epidemiol Infect.

[27] Moosavian M, Ghaderiyan GH, Shahin M, Navidifar T (2019). First investigation of the presence of SPATE genes in *Shigella* species isolated from children with diarrhea infection in Ahvaz, southwest Iran. Infection and Drug Resistance.

[28] Salah M, Shtayeh I, Ghneim R, Al-Qass R, Sabateen A, Marzouqa H (2019). Evaluation of *Shigella* species azithromycin CLSI epidemiological cutoff values and macrolide resistance genes. J Clin Microbiol.

[29] Williams P, Berkley J (2016). Dysentery (Shigellosis) current WHO guidelines and the WHO essential” medicine list for children.

[30] Kang H, Wang L, Li Y, Lu Y, Fan W, Bi R (2019). Dissemination of multidrug-resistant *Shigella flexneri* and *Shigella sonnei* with class 1, class 2, and atypical class 1 integrons in China. Microb Drug Resist.

[31] Ud-Din AI, Wahid SU, Latif HA, Shahnaij M, Akter M, Azmi IJ (2013). Changing trends in the prevalence of *Shigella* species: emergence of multi-drug resistant *Shigella sonnei* biotype g in Bangladesh. PLoS ONE.

[32] Chattaway MA, Schaefer U, Tewolde R, Dallman TJ, Jenkins C (2017). Identification of *Escherichia coli* and *Shigella* species from whole-genome sequences. J Clin Microbiol.

[33] Bielaszewska M, Idelevich EA, Zhang W, Bauwens A, Schaumburg F, Mellmann A (2012). Effects of antibiotics on Shiga toxin 2 production and bacteriophage induction by epidemic *Escherichia coli* O104:H4 strain. Antimicrob Agents Chemother.

[34] Lin Z, Cai X, Chen M, Ye L, Wu Y, Wang X (2018). Virulence and stress responses of *Shigella flexneri* regulated by PhoP/PhoQ. Front Microbiol.

[35] Dowd SE, Killinger-Mann K, Brashears M, Fralick J (2008). Evaluation of gene expression in a single antibiotic exposure-derived isolate of *Salmonella enterica* typhimurium 14028 possessing resistance to multiple antibiotics. Foodborne Pathog Dis.

[36] Sharma AK, Payne SM (2006). Induction of expression of hfq by DksA is essential for *Shigella flexneri* virulence. Mol Microbiol.

[37] Sharma AK. Role of DksA and Hfq in *Shigella flexneri* virulence. 2007.

[38] Hu N, Qian L, Hu Y, Shou J-Z, Wang C, Giffen C (2006). Quantitative real-time RT-PCR validation of differential mRNA expression of SPARC, FADD, Fascin, COL7A1, CK4, TGM3, ECM1, PPL and EVPLin esophageal squamous cell carcinoma. BMC Cancer.

[39] Koppolu V, Osaka I, Skredenske JM, Kettle B, Hefty PS, Li J (2013). Small-molecule inhibitor of the *Shigella flexneri* master virulence regulator VirF. Infect Immun.

